# Two freshwater shrimp species of the genus *Caridina* (Decapoda, Caridea, Atyidae) from Dawanshan Island, Guangdong, China, with the description of a new species

**DOI:** 10.3897/zookeys.923.48593

**Published:** 2020-04-01

**Authors:** Qing-Hua Chen, Wen-Jian Chen, Xiao-Zhuang Zheng, Zhao-Liang Guo

**Affiliations:** 1 South China Institute of Environmental Sciences, Ministry of Ecology and Environment, Guangzhou 510520, Guangdong Province, China South China Institute of Environmental Sciences, Ministry of Ecology and Environment Guangzhou China; 2 Department of Animal Science, School of Life Science and Engineering, Foshan University, Foshan 528231, Guangdong Province, China Foshan University Foshan China

**Keywords:** COI, morphology, rice shrimp, systematics, taxonomy

## Abstract

A faunistic and ecological survey was conducted to document the diversity of freshwater atyid shrimps of Dawanshan Island. Two species of *Caridina* that occur on this island were documented and discussed. One of these, *Caridina tetrazona***sp. nov.** is described and illustrated as new to science. It can be easily distinguished from its congeners based on a combination of characters, which includes a short rostrum, the shape of the endopod of the male first pleopod, the segmental ratios of antennular peduncle and third maxilliped, the slender scaphocerite, and the absence of a median projection on the posterior margin. Live individuals of the new species display a unique coloration pattern with four dark blue transverse bands on the body, and can be easily recognized in the field. So far, despite considerable surveying efforts made on neighboring islands, this species has only been found from a small stream on Dawanshan Island, which suggests that it may have a very limited range, probably endemic to Dawanshan Island. Molecular characteristics of the mitochondrial cytochrome c oxidase subunit I (COI) demonstrate that this species shows sufficient interspecific divergence from its congeners, including *C. serrata* Stimpson, 1860, which was found in four streams on Dawanshan Island, and has been previously reported on the neighboring islands of Hong Kong, Dong’ao, Wailingding, and Guishan.

## Introduction

The genus *Caridina* H. Milne Edwards, 1837, comprising 302 species and mainly present in the Indo-Pacific region, is the most diversified genus of the Atyidae (De Grave et al. 2015; De Mazancourt et al. 2018). Collecting evidence-based information is the foundation for addressing urgent global challenges in biodiversity conservation and sustainable management of native species. The critical knowledge gaps in the taxonomy, population and distribution patterns have to be filled before any decisions can be taken on biologically meaningful and effective conservation management. The atyid fauna of Dawanshan Island has not been properly surveyed. In order to better understand the diversity of the freshwater atyid fauna in the Dawanshan Island, an intensive field survey was carried out in June 2017. The results show that there are two species of atyid shrimps, *Caridina tetrazona* sp. nov. and *C. serrata* Stimpson, 1860.

**Figure 1.**
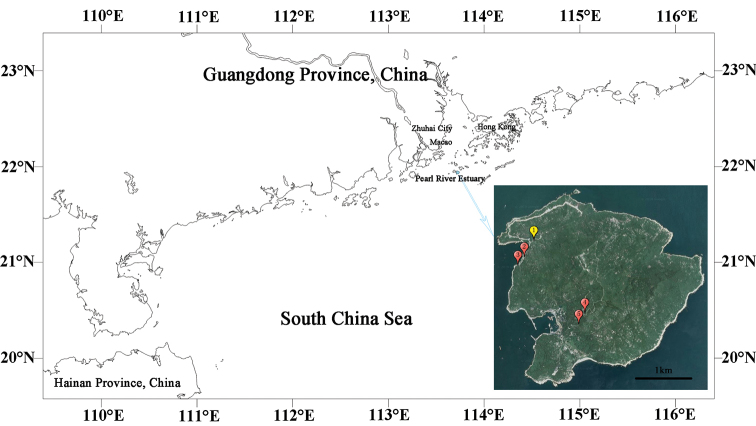
The light blue point shows the location of Dawanshan Island. The inset shows the sample locations. The yellow icon (stn 1) shows the collection site of *Caridina tetrazona* sp. nov., and red icons (stns 2–5) are the collection sites of *C. serrata*.

The Wanshan Islands are located in the Pearl River Estuary, Zhuhai, Guangdong Province, southern China. Dawanshan Island (21°55'19.69" – 21°57'21.46"N, 113°42'54.30" – 113°45'06.09"E) covers an area of 8175 km^2^, and is situated in the south of the Wanshan Islands. It is about 29 km northwest from Macau, 33 km from Zhuhai City, and 56 km northeast from Hong Kong (Fig. 1).

With an unspoiled natural landscape and an ideal climate, the island is promising for marine ecotourism development. The increasing exploitation of resources for tourism threatens the species that live there. The new species could be potentially seriously threatened and should be regarded as an endangered species.

## Material and methods

### Study area

Dawanshan Island (21°55'19.69" – 21°57'21.46"N, 113°42'54.30" – 113°45'06.09"E) belongs to the Wanshan Islands. It is a small island, 3.35 km in length, 2.45–3.88 km in width, with a coastline of 14.42 km, and five bays around the island. There are five peaks, with the highest point of Wanshan Peak in the central part of the north, at 432.5 m above sea level. Dawanshan Island has loess sandy soil over a rocky base. There are many cliffs on both sides of the south and west, and huge rocks on both sides of the east and north. The island has a subtropical oceanic monsoon climate, which is warm and humid throughout the year, with an average annual temperature of 22–23 °C, an average annual precipitation of 1800–2000 mm and an average annual relative humidity of 81.0%. Vegetation on Dawanshan Island is of evergreen broad-leaved forest type, with a forest coverage rate of about 60% (Huang and Wang 2000; Liao et al. 2000; He et al. 2004; Huang et al. 2012). The sampling sites of the current study, covering habitats of streams, pools, agricultural waterways, swamps, and brackish water bays are provided in Figure 1.

### Collection

Samples were collected by a hand net with a mesh size of 0.8 mm. All specimens obtained were fixed in 95% ethanol. The ethanol was changed after 24 hours with fresh 75% ethanol. The drawings were made with the aid of a drawing tube mounted on an Olympus BX – 41 compound microscope.

### Genetic analyses

The forward and reverse primers of the mitochondrial COI gene in this study were: LCO1490 and HCO2198, respectively (Folmer et al. 1994). The PCR reaction was carried out on a Bio-Rad/T^100^TM Thermal Cycler instrument with a system of 50 μL, of which 25 μL of 2 × EasyTaq Mix, 20 μL of ddH_2_O, 2 μL of each of the forward and reverse primers, and 1 μL of the DNA template. The PCR amplification conditions were: 35 cycles of denaturation at 94 °C for 30 s, annealing at 46 °C for 60 s, extension at 72 °C for 60 s, and a final extension at 72 °C for 5 min. Five μL of the PCR product was subjected to 1.5% agarose gel electrophoresis for detection of the amplified product. After successful detection, sequences were obtained by Applied Biosystems 3730 Analyzer (Applied Biosystems, Foster City, CA, USA), after verification with the complementary strand.

A total of 26 nucleotide sequences were analyzed. Selected species for the molecular analyses were species similar to the morphology and the color of the new species and the *Caridina* species that are known to occur on neighbouring islands. All the sequences were aligned with MAFFT v7.313 software using the auto strategy and normal alignment mode (Katoh and Standley 2013). In order to find the best-fitting model for sequence evolution, ModelFinder (Kalyaanamoorthy et al. 2017) was used. According to Bayes information criterion (ModelFinder default recommendation), the best models for analysis maximum likelihood method (ML) and Bayesian method (BI) are TIM2+F+I+G4 and GTR+F+G4, respectively. ML was performed using IQ-TREE 1.6.12 (Nguyen et al. 2015). Bayesian phylogeny was applied using MrBayes v3.2.6 (Ronquist et al. 2012). Markov chain Monte Carlo analysis was performed with two simultaneous runs starting with random tress to approximate the posterior probabilities of trees. Each run consisted of four chains, with default heating parameters. It lasted for 2 × 10^6^ generations for the selected Atyidae and the first 25% of the samples were discarded. Standard deviation of split frequencies (<0.01) was accounted as a convergence index. Genetic distances were calculated using the Kimura 2-parameter model in MEGA 7.0 based on COI (Kumar et al. 2016).

**Table 1.** Specimens of the atyids *Caridina* and *Neocaridina* used in the molecular analyses (new sequences), listed by localities, geographical coordinates and GenBank accession numbers.

Species	Locality	Geographical coordinates	Accession no.
*C. cantonensis*	Wutong Mountain, Shenzhen	22°34'49"N, 114°12'44"E	MN701589
	Wutong Mountain, Shenzhen	22°34'49"N, 114°12'44"E	MN701590
	Baiyun Mountain, Guangzhou	23°10'05"N, 113°17'36"E	MN701591
	Baiyun Mountain, Guangzhou	23°10'05"N, 113°17'36"E	MN701592
*C. huananensis*	Yingde, Qingyuan	23°54'17"N, 113°15'55"E	MN701607
	Yingde, Qingyuan	23°54'17"N, 113°15'55"E	MN701608
*C. lanceifrons*	Dongfang, Hainan	18°40'06"N, 109°56'32"E	MN701605
	Dongfang, Hainan	18°40'06"N, 109°56'32"E	MN701606
*C. mariae*	Nankun Mountain, Huizhou	23°39'32"N, 113°54'19"E	MN701601
	Nankun Mountain, Huizhou	23°39'32"N, 113°54'19"E	MN701602
*C. serrata*	Dawanshan Island, Zhuhai	21°33'41"N, 113°25'59"E	MN701595
	Dawanshan Island, Zhuhai	21°33'41"N, 113°25'59"E	MN701596
	Dong’ao Island, Zhuhai	22°00'17"N, 113°25'27"E	MN701599
	Dong’ao Island, Zhuhai	22°00'17"N, 113°25'27"E	MN701600
*C. tetrazona* sp. nov.	Dawanshan Island, Zhuhai	21°33'57"N, 113°25'48"E	MN701593
	Dawanshan Island, Zhuhai	21°33'57"N, 113°25'48"E	MN701594
*C. zhujiangensis*	Dong’ao Island, Zhuhai	22°00'38"N, 113°25'13"E	MN701603
	Dong’ao Island, Foshan	22°00'38"N, 113°25'13"E	MN701604
*N. palmata*	Yangshan, Qingyuan	24°25'28﻿"N, 112°36'28"E	MN701609
	Yangshan, Qingyuan	24°25'28"N, 112°36'28"E	MN701610
*N. hofendopoda*	Sanxia, Yichang	30°49'32"N, 111°00'59"E	MN701611
	Sanxia, Yichang	30°49'32"N, 111°00'59"E	MN701612

### Abbreviations

The following abbreviations are used throughout the text: al: altitude; cl: carapace length (measured from the postorbital margin to the posterior margin of the carapace); coll: sample collectors; rl: rostral length (measured from the rostral tip to the postorbital margin); stn: sampling station; tl: total length (measured from the rostral tip to the posterior margin of the telson). All measurements are in millimeters.

Specimens were deposited in the Department of Animal Science, School of Life Science and Engineering, Foshan University (FU).

## Systematic accounts

### Family Atyidae De Haan, 1849

**Subfamily Atyinae De Haan, 1849**

**Genus *Caridina* H. Milne Edwards, 1837**

#### Caridina serrata

Taxon classificationAnimaliaDecapodaAtyidae

Stimpson, 1860

2FE57FAC-DC28-5D86-A1F8-60EDCE685663

Figs 2B, C , 3B, C

Caridina serrata Stimpson, 1860: 29 [type locality: Hong Kong, China].
Caridina serrata -Ortmann 1894: 406; Bouvier 1905: 76; 1925: 258, fig. 593; Kemp 1918: 289, fig. 12; Cai and Ng 1999: 1605, figs 2, 3; Liang 2004: 173, fig. 83; Chen et al. 2018: 315, figs 2, 3.

##### Material examined.

13 females, cl 3.5–6.8 mm, 2 ovigerous cl 3.7–5.6 mm, 5 males cl 3.0–5.8 mm, a small pool (21°56'48.6"N, 113°42'55.5"E, al. 5.4 m, stn 2), 27 June 2017, coll. Z. L. Guo, W. J. Chen; 17 females, cl 3.4–6.6 mm, 2 ovigerous females, cl 4.1–5.6 mm, 15 males, cl 2.9–5.5 mm, a small stream (21°56'43.6"N, 113°42'51.4"E, al. 27.2 m, stn 3), 27 June 2017, coll. Z. L. Guo, W. J. Chen; 5 females, cl 3.1–6.9 mm, 3 ovigerous, cl 3.5–5.8 mm, 3 males, cl 3.0–5.2 mm, a small stream (21°56'15.6"N, 113°43'33.4"E, al. 186.6 m, stn 4), 28 June 2017, coll. Z. L. Guo, W. J. Chen; 7 females, cl 3.4–6.9 mm, 4 males, cl 3.5–5.7 mm, a small stream (21°56'02.8"N, 113°43'29.0"E, al. 122.8 m, stn 5), 28 June 2017, coll. Z. L. Guo, W. J. Chen.

##### Remarks.

The present specimens are in agreement with the description and illustrations of Cai and Ng (1999) and Liang (2004). *Caridina serrata* is highly adaptable and prolific, distributed everywhere on the Wanshan Islands, such as Dong’ao Island, Guishan Island, and Wailingding Island (Chen et al. 2018). A close biogeographical connection of the atyid faunas among the Wanshan Islands is evident. This species is also distributed in Hong Kong (Cai and Ng 1999), Chaqiao, and Zhongshan City (Liang 2004).

##### Ecological notes.

**Figure 2.**
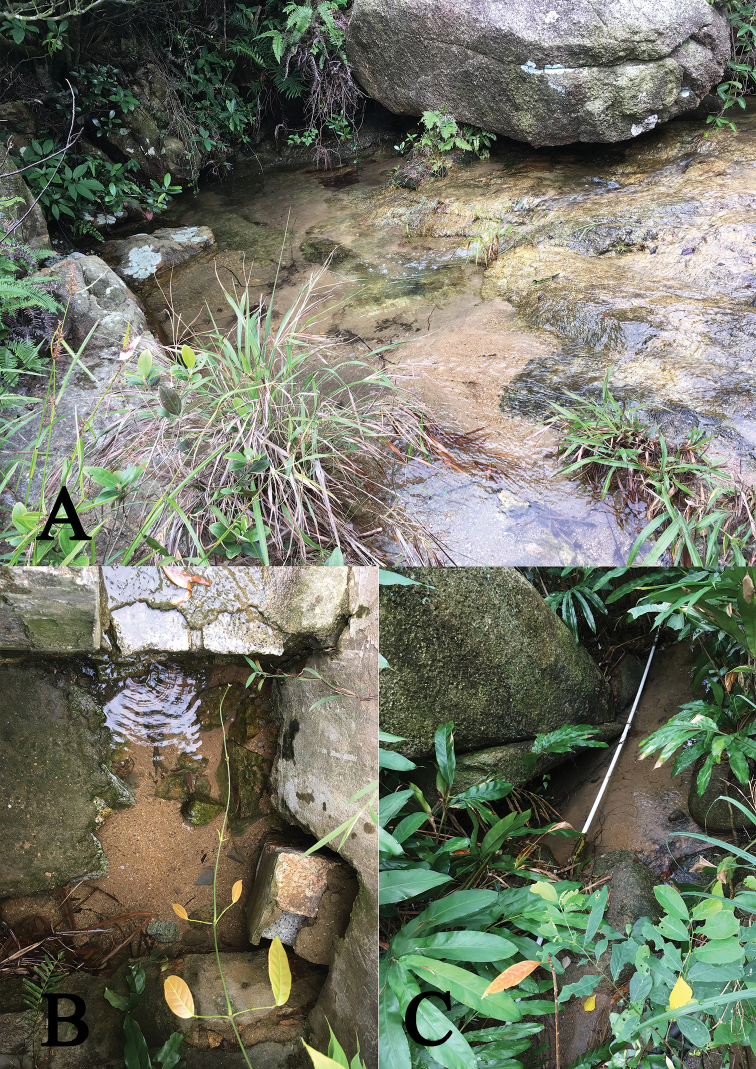
Habitats of the two shrimp species in this study. **A** Habitat of *Caridina tetrazona* sp. nov. **B, C** habitat of *C. serrata*.

*Caridina serrata* is commonly found in pools, streams, and artificial ditches on the island. Sometimes, the stream connects with the sea. Sediment at the site comprised sand, pebbles and gravel patches between large boulders. Hill streams are within secondary forests and are covered with aquatic plant (Fig. 2B, C). The water parameters of the streams at the time of collection were: temperature 24–26 °C, pH 5.8–6.5, dissolved ammonia nitrogen 0.20–0.22 mgl^-1^, and dissolved oxygen 7.8–8.6 mgl^-1^; the water was clear and flowing. *Caridina serrata* is associated with dead leaves and aquatic plants, but also lives under pebbles and stones. The female carries fewer but large eggs (0.5–0.7 × 0.9–1.0 mm). The larvae go through direct development and hatch into benthic hatchlings that resemble miniature adults.

##### Coloration.

**Figure 3.**
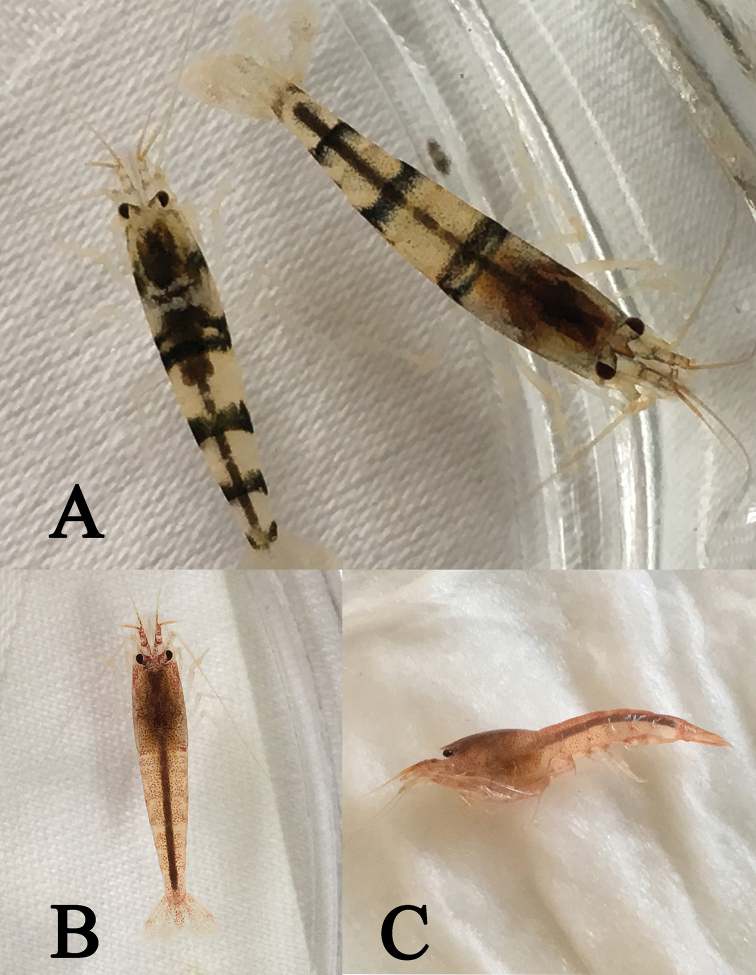
Photos of live specimens of the two species of *Caridina* from Dawanshan Island. **A***Caridina tetrazona* sp. nov. **B** dorsal view of *C. serrata***C** lateral view of *C. serrata*.

The live shrimp show light-red coloration and are translucent (Fig. 3B, C).

##### Distribution.

Southern China (Hong Kong, Zhuhai City and Zhongshan City of Guangdong Province).

#### Caridina tetrazona sp. nov.

Taxon classificationAnimaliaDecapodaAtyidae

3F0711A4-4E95-51CF-AF4E-9E5E8EE93B51

http://zoobank.org/F3E1596D-A5CE-47B0-8926-19E95DAAC97A

**Figure 4.**
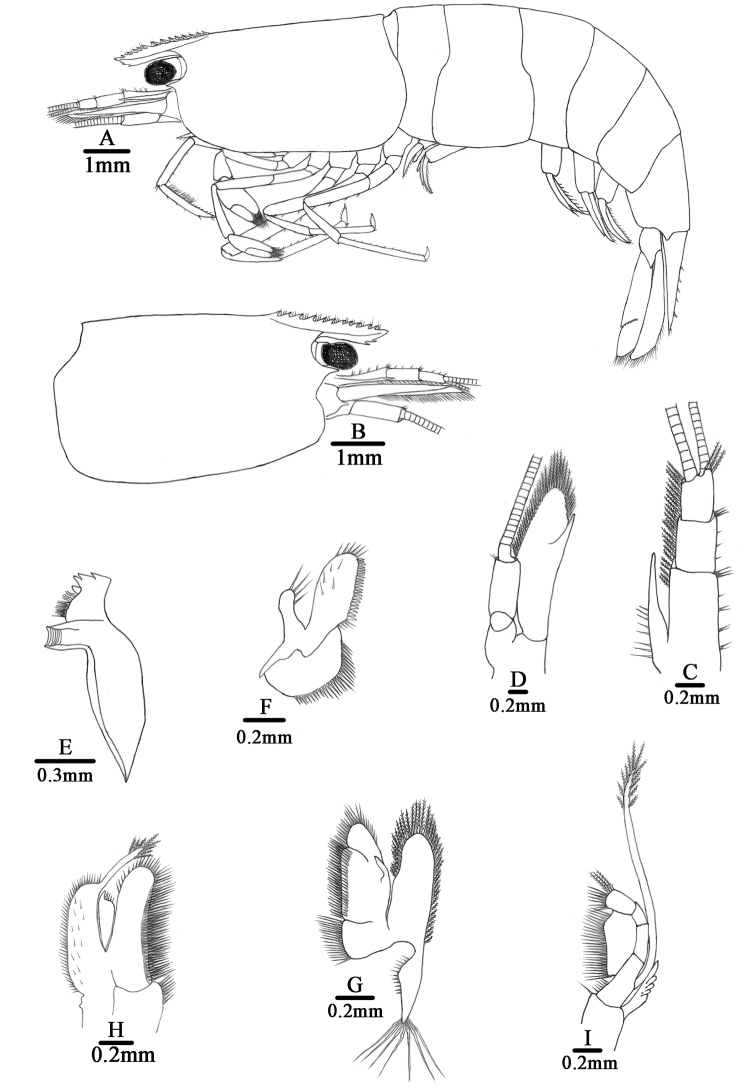
*Caridina tetrazona* sp. nov. **A** Entire animal, lateral view, holotype (FU, 2017-06-27-01) **B–I** paratype (FU, 2017-06-27-02) **B** carapace and cephalic appendages, lateral view **C** antennule **D** antenna **E** mandible **F** maxillula **G** maxilla **H** first maxilliped **I** second maxilliped.

**Figure 5.**
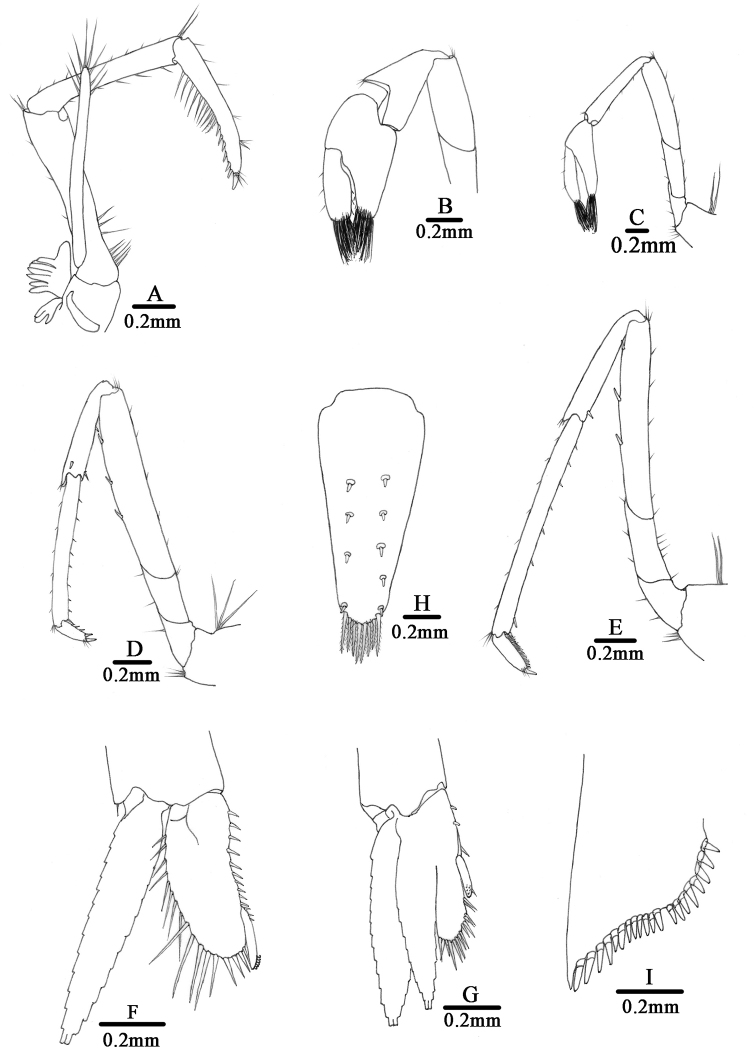
*Caridina tetrazona* sp. nov. paratype (FU, 2017-06-27-02). **A** Third maxilliped **B** first pereiopod **C** second pereiopod **D** third pereiopod **E** fifth pereiopod **F** first pleopod **G** second pleopod **H** telson **I** diaeresis of uropodal exopod.

Figs 2A , 3A , 4 , 5

##### Material examined.

***Holotype***: male (FU, 2017-06-27-01), cl 3.9 mm, tl 14.4 mm, rl 1.3 mm, a stream near Longtangzui Dawanshan Island, Zhuhai City, Guangdong, China (21°56'59.2"N, 113°43'00"E, al. 8 m, stn 1), 9 June 2017. ***Paratypes***: male (FU, 2017-06-27-02), cl 4.3 mm, 4 males (FU, 2017-06-27-03), cl 3.8–4.3 mm, 33 females, 4 ovigerous (FU, 2017-06-27-04), cl 3.8–5.4 mm, same collection data as for holotype, coll. Z. L. Guo, W. J. Chen.

##### Comparative material.

*Caridina serrata* Stimpson, 1860 (see material under *Caridina serrata*). *Caridina cantonensis* Yu, 1938; 8 males, cl 3.5–6.3 mm, 5 females, 2 ovigerous, cl 4.1–6.3 mm, a stream at Mangzixia, Yingde, Qingyuan City, Guangdong, China (24°3'20"N, 113°19'6"E, al. 20 m), 4 June 2018, coll. Z. L. GUO, W. J. Chen, X. Z. Zheng.

##### Diagnosis.

Rostrum short, straight or slightly sloping downwards, nearly reaching to or slightly reaching beyond end of 1^st^ segment of antennular peduncle, rostral formula 3–8 (usually 5–7) + 4–6/1–2. 1^st^ pereiopod carpus 0.63–0.70 × as long as chela, 1.6–1.7 × as long as high; chela 1.8–2.0 × as long as broad; fingers 0.92–1.1 × as long as palm. 2^nd^ pereiopod carpus 1.1–1.3 × as long as chela, 4.9–5.3 × as long as high; chela 2.7–2.9 × as long as broad; fingers 1.7–1.8 × as long as palm. 3^rd^ pereiopod propodus 3.9–4.5× as long as dactylus, with 8–11 thin spines on the posterior and lateral margins. 5^th^ pereiopod propodus 4.9–5.1 × as long as dactylus, with 8–11 thin spines on the posterior and lateral margins, dactylus terminating in one claw, with 27–31 spinules on flexor margin. Endopod of male 1^st^ pleopod extending to 0.56 × exopod length, wider proximally, subrectangular, 2.5–2.6 × as long as wide, appendix interna well developed, arising from distal 1/3 of endopod, reaching beyond end of endopod. Appendix masculina of male 2^nd^ pleopod rod-shaped, reaching to 0.7 length of endopod, appendix interna reaching to 0.7 length of appendix masculina. Uropodal diaeresis with 18–20 movable spinules. Eggs 0.51–0.65 × 0.84–0.97 mm in diameter.

##### Description.

Body: small, slender and sub-cylindrical, males up to 14.8 mm tl, females up to 20.4 mm tl.

Rostrum (Fig. 4A, B): Short, only 0.15–0.32 of cl, straight or slightly sloping downwards; reaching end of basal segment of antennular peduncle, or to just beyond it; armed dorsally with 7–14 teeth, including 3–8 (usually 5–7) on carapace posterior to orbital margin, ventrally with 1–2 teeth; lateral carina dividing rostrum into two unequal parts, continuing posteriorly to orbital margin.

Eyes (Fig. 4A, B): Well developed on short ocular peduncle, cornea globular.

Carapace (Fig. 4A, B): Smooth, glabrous; antennal spine acute, fused with inferior orbital angle; pterygostomian margin broadly rounded or slightly produced forward; no pterygostomian.

Antennule (Fig. 4A–C): Peduncle reaching slightly short of scaphocerite; stylocerite long, reaching 0.3 × of 2^nd^ segment; anterolateral angle reaching 0.2 × of 2^nd^ segment; length of basal segment as long as sum of length of 2^nd^ and 3^rd^ segments, 2^nd^ segment 0.46–0.51 × of basal segment, 1.1–1.3 × of 3^rd^ segment; all segments with sub-marginal plumose setae.

Antenna (Fig. 4D): Peduncle about 0.54 × of scaphocerite; scaphocerite about 3.6 × as long as wide, outer margin straight, ending in strong sub-apical spine, inner and anterior margins with long plumose setae.

Mandible (Fig. 4E): Without palp; left incisor process with 4 sharp teeth; medially 2 groups of setae; molar process ridged.

Maxillula (Fig. 4F): Lower lacinia broadly rounded, with several rows of plumose setae; upper lacinia elongate, medial edge straight, with 29 strong spinules and simple setae; palp simple, slightly expanding distally, with 4 long simple setae.

Maxilla (Fig. 4G): Scaphognathite tapering posteriorly, distally with regular row of long plumose setae and short marginal plumose setae continuing down proximal triangular process, furnished with numerous long plumose setae; upper and middle endite with marginal simple, denticulate and sub-marginal simple setae, distally with plumose setae; lower endite with long simple marginal setae; palp distinctly shorter than cleft of upper endite, wider proximally than distally.

First maxilliped (Fig. 4H): Palp broadly triangular ending in fringe-like tip and with terminal plumose setae; caridean lobe broad, with marginal plumose setae; exopodal flagellum well developed, with distally marginal plumose setae; ultimate and penultimate segments of endopod indistinctly divided; medial and distal margins of ultimate segment with marginal and sub-marginal rows of simple, denticulate and plumose setae; penultimate segments with marginal long plumose setae.

Second maxilliped (Fig. 4I): Ultimate and penultimate segments of endopod indistinctly divided, reflexed against basal segment; inner margin of ultimate, penultimate and basal segments with long setae of various types; exopod flagellum long, slender with marginal plumose setae distally.

Branchial formula typical for genus. Epipod on first four pereiopods.

Third maxilliped (Fig. 5A): Reaches to end of 2^nd^ antennular peduncle segment, endopod three-segmented, penultimate segment as long as basal segment; distal segment 0.87–0.90 × as long as penultimate segment, ending in a large claw-like spine surrounded by simple setae, preceded by about 5–8 spines on distal third of posterior margin, proximally a clump of long and short simple, serrate setae; exopod reaches to end of basal segment of endopod, distal margin with long plumose setae.

First pereiopod (Fig. 5B): Reaches end of eyes; chela 1.8–2.2 × as long as wide; 1.4–1.6 × as long as carpus; movable finger 2.8–3.2 × as long as wide, 0.92–1.1× as long as palm, setal brushes well developed; carpus excavated anterior-dorsally, 1.5–1.8 × as long as high, 0.90–1.0 × as long as merus.

Second pereiopod (Fig. 5C): Reaches about end of 3^rd^ antennular peduncle segment, more slender and longer than first pereiopod; chela 2.6–3.2 × as long as wide; 0.78–0.87 × as long as carpus; movable finger 4.0–4.6 × as long as wide and 1.5–1.7 × as long as palm, setal brushes well developed; carpus 4.9–5.9 × as long as high, slightly excavated anterior, as long as merus.

Third pereiopod (Fig. 5D): Reaches beyond end of scaphocerite; dactylus 2.9–3.5 × as long as wide, ending in prominent claw-like spine surrounded by simple setae, flexor margin bearing 4–5 spines; propodus 4.5–4.9 × as long as dactylus, bearing 8–12 spinules on posterior and lateral margin, 8.4–9.7 × as long as wide; carpus 0.67–0.82 × as long as propodus; merus 1.7–2.0× as long as carpus, with about 3–4 strong spines on the posterior margin.

Fourth pereiopod: Reaches middle of 2^nd^ segment of antennular peduncle; dactylus 3.0–4.2 × as long as wide, ending in prominent claw-like spine surrounded by simple setae, flexor margin bearing 4–6 spines; propodus 4.2–5.1 × as long as dactylus, bearing 9–13 spinules on posterior and lateral margin, 8.5–10.5 × as long as wide; carpus 0.66–0.81 × as long as propodus; merus 1.6–1.8× as long as carpus, with about 3–4 strong spines on the posterior margin.

Fifth pereiopod (Fig. 5E): Reaches middle of 1^st^ segment of antennular peduncle; dactylus 2.8–3.6 × as long as wide, ending in prominent claw-like spine surrounded by simple setae, flexor margin bearing with a row of 27–31 comb-like spinules; propodus 4.7–5.1 × as long as dactylus, bearing 8–11 spinules on posterior and lateral margin, 8.9–11.9 × as long as wide; carpus 0.52–0.62 × as long as propodus; merus 1.3–1.5 × as long as carpus, with about 3–4 strong spines on the posterior margin.

First pleopod (Fig. 5F): Endopod of male subrectangular, wider proximally, 0.56 × as long as exopod, 2.5–2.6 × as long as proximal wide, ending broadly rounded; inner margin slightly concave, bearing short spine-like setae, outer margin slightly convex, bearing long spine-like setae, distinctly longer and stout on distal 1/3, distal end tip bearing nearly equal length shorter spine-like setae; appendix interna well developed, arising from distal 1/3 of endopod, reaching beyond end of endopod, distally with numerous cincinulli.

Second pleopod (Fig. 5G): Appendix masculina rod-shaped, reaching about 0.7 × length of exopod, inner margin and tip bearing numerous spine setae; appendix interna slender, reaching about 0.73 × length of appendix masculina, distally with many cincinulli.

Telson (Fig. 5H): 0.56–0.75 × as long as cl, tapering posterior, dorsal surface with 6 pairs of stout movable spine-like setae including the pair at poster lateral angles; posterior margin with 4–5 pairs of intermediate plumose setae, the outer one usually strongest and longest, no median projection on posterior margin. Exopodite of uropod bearing a series of 18–20 movable spinules on the diaeresis.

Eggs 0.51–0.65 × 0.84–0.97 mm in diameter.

Coloration: Body semi-translucent, four dark blue bands transverse on the tergum of the 1^st^, 3^rd^, 5^th^, and 6^th^ abdominal segments, appendages mostly translucent (Fig. 3A).

##### Etymology.

*Caridina tetrazona* is a combination of two Latin words, *tetra*, four, and *zona*, band, alluding to its four-banded color pattern.

##### Remarks.

*Caridina tetrazona* sp. nov. is most similar to *C. serrata* Stimpson, 1860 (which also occurs on the same island) in the short rostrum, the shape of the endopod of the male first pleopod, and the similar-sized eggs. It can be easily distinguished from *C. serrata* by the length of the basal segment of the antennular peduncle which is as long as the combined length of 2^nd^ and 3^rd^ segments (versus length of basal segment distinctly longer than the combined length of 2^nd^ and 3^rd^ segments in *C. serrata*); length of penultimate segment of 3^rd^ maxilliped as long as basal segment; the distal segment is distinctly shorter than the penultimate segment (versus penultimate segment distinct shorter than basal segment and distal segment as long as penultimate segment in *C. serrata*); the absence of a projection on the posterior margin of telson (versus with a projection in *C. serrata*), and the slender scaphocerite (3.6–3.7 times as long as wide versus 3.0–3.2 times in *C. serrata*). In addition, the four dark blue bands on the body of live shrimps allow an easy identification in the field. *Caridina tetrazona* sp. nov. also shows close similarity with *C. cantonensis* Yu, 1938 regarding the ratios of various segments of the 1^st^ and 2^nd^ pereiopods. Beside its peculiar coloration, *C. tetrazona* sp. nov. differs from *C. cantonensis* in the short rostrum, which only reaches the end of 1^st^ segment of the antennular peduncle (versus distinctly reaches beyond the end of the 2^nd^ segment in *C. cantonensis*); the endopod of the male first pleopod slender (2.6–2.7 times as long as wide versus 2.1–2.4 times in *C. cantonensis*) and distal part not dilated (versus distally distinctively dilated in *C. cantonensis*); palp of 1^st^ maxilliped ending in a finger-like tip (versus broadly rounded in *C. cantonensis*); and absence of a projection on the posterior telsonic margin (versus with a projection in *C. cantonensis*). *Caridina tetrazona* sp. nov. closely resembles *C. trifasciata* Yam & Cai, 2003, in having similar dark blue bands on their abdomen, and in having the shape of the endopod of the male 1^st^ pleopod and appendix masculina of the male 2^nd^ pleopod similar. *Caridina tetrazona* sp. nov. differs from *C. trifasciata* in its proportionately shorter rostrum (only reaches the end of the 1^st^ segment of the antennular peduncle versus reaches beyond the end of the 2^nd^ segment in *C. trifasciata*); palp of the 1^st^ maxilliped ending in a finger-like tip (versus broadly rounded in *C. trifasciata*); and the slender scaphocerite (3.6–3.7 times as long as wide versus 2.8 times in *C. trifasciata*).

##### Ecological notes.

The type specimens were collected from a small stream at an elevation of 8 m, stn 1, near Longtangzui, Dawanshan Island, Zhuhai City, Guangdong, China (21°56'59.2"N, 113°43'00"E) (Fig. 1). The stream is 2–3 m in width and 0.3–0.5 m in depth. The stream bed consists of rocks interspersed with gravel and sands patches. The bank was covered with aquatic vegetation. The shrimps live among rocks and marginal vegetation. The stream water was fast flowing and the temperature was 26 °C, pH was 6.4, and dissolved oxygen concentrations was 7.8 mg/1.

##### Distribution.

only known from Guangdong Province, southern China.

## Molecular phylogenetic results

**Figure 6.**
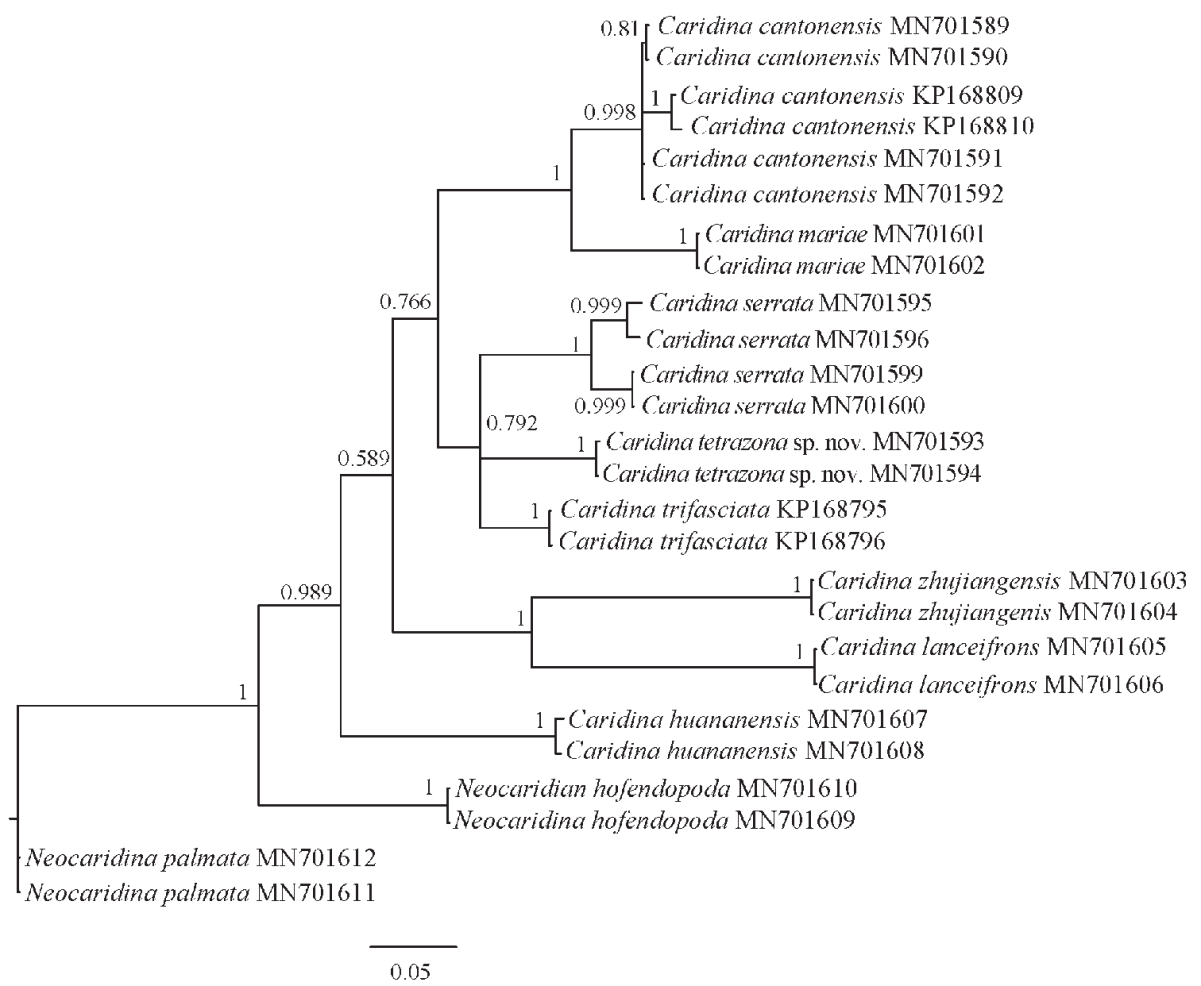
Bayesian inference tree of species of *Caridina* and outgroups (*Neocaridina*) based on COI gene. Support values at the nodes represent posterior probability.

**Figure 7.**
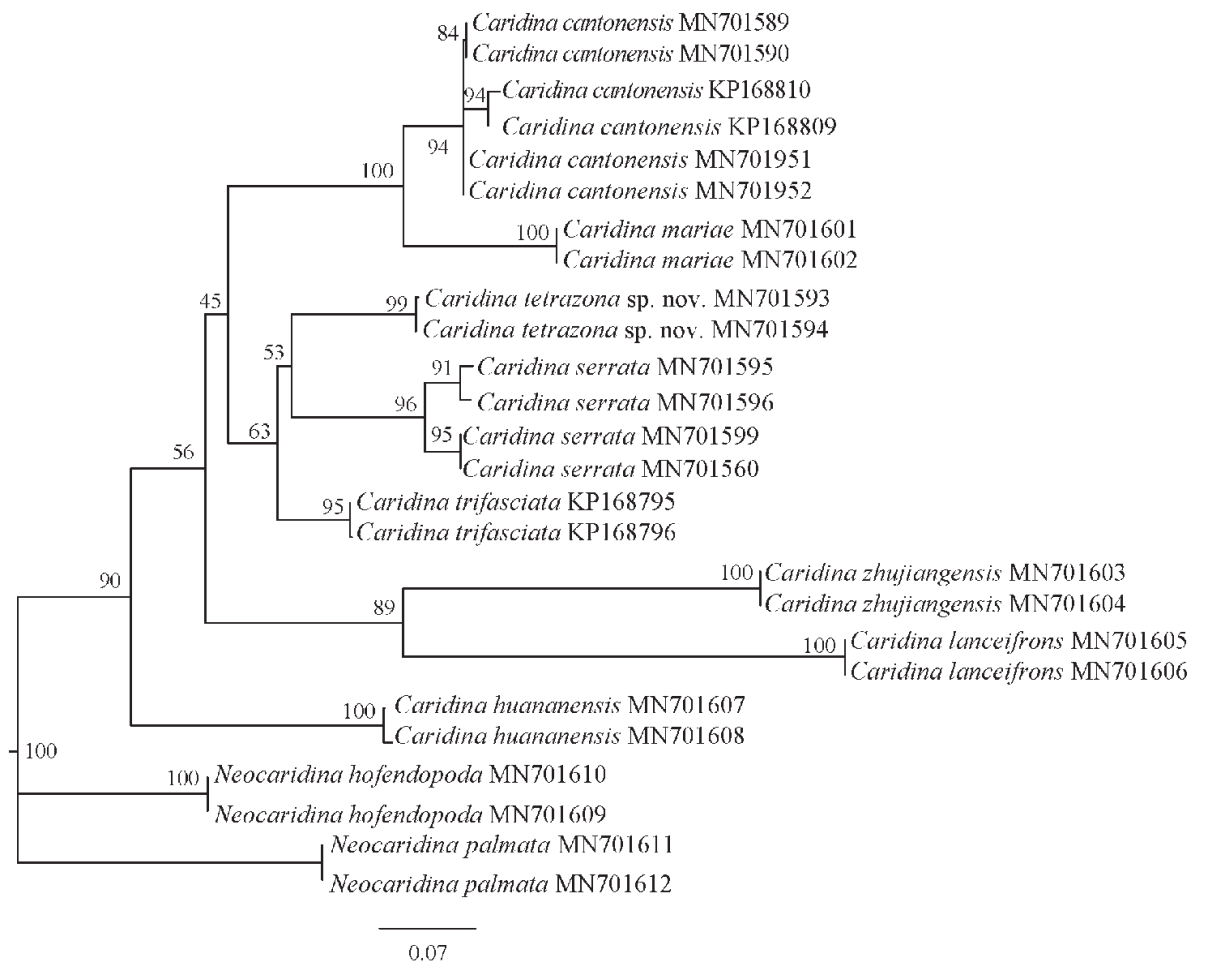
Phylogenetic tree (ML) based on COI gene fragments of 10 shrimp species of *Caridina* and *Neocaridina* (outgroups). Support values at the nodes represent bootstrap values for ML.

The primers used in this study are located at the 5’ end of the COI gene, and the new sequencing results are corrected for 624~bp for subsequent analysis. As can be seen from Table 2, pairwise genetic distances between *Caridina tetrazona* sp. nov. and *C. serrata* and *C. cantonensis* are 0.067 and 0.128, respectively. The topology of the Bayesian (BI) trees and the ML tree are basically similar. Phylogenetic trees revealed the relationship between *Caridina tetrazona* sp. nov. and nine other species of Atyidae, with the posterior probability and bootstrap values from the BI and ML analyses shown in Figures 6 and 7.

**Table 2.** Pairwise genetic distance among eight *Caridina* species (Atyidae) based on the COI gene. The range of genetic distances between different species is in parentheses.

Species	1	2	3	4	5	6	7
**1. *C. cantonensis***							
**2. *C. huananensis***	0.189 (0.171–0.207)						
**3. *C. lanceifrons***	0.234 (0.221–0.246)	0.246 (0.245–0.246)					
**4. *C. mariae***	0.110 (0.100–0.119)	0.216 (0.215–0.216)	0.277 (0.277–0.277)				
**5. *C. serrata***	0.149 (0.139–0.159)	0.240 (0.224–0.255)	0.260 (0.252–0.268)	0.144 (0.137–0.150)			
**6. *C. tetrazona* sp. nov.**	0.128 (0.118–0.138)	0.203 (0.199–0.206)	0.227 (0.227–0.227)	0.137 (0.137–0.137)	0.067 (0.033–0.101)		
**7. *C. trifasciata***	0.112 (0.105–0.118)	0.214 (0.206–0.221)	0.256 (0.252–0.260)	0.147 (0.144–0.150)	0.117 (0.100–0.134)	0.091 (0.088–0.094)	
**8. *C. zhujiangensis***	0.237 (0.229–0.244)	0.325 (0.321–0.329)	0.265 (0.265–0.265)	0.281 (0.281–0.281)	0.291 (0.279–0.303)	0.263 (0.263–0.263)	0.241 (0.237–0.245)

## Discussion

The faunistic and ecological results of the present survey illustrate that the freshwater atyid shrimps are not very diverse along the habitats of the coastline of Dawansha Island; only two species of *Caridina* occur on this island. *Caridina tetrazona* is a new species, while *Caridina serrata* Stimpson, 1860 is known from a wide area and can be commonly found in streams from this island. In addition, it also occurs on neighboring islands, i.e., Hong Kong, Dong’ao Island, Wailingding Island, and Guishan Island (Cai and Ng 1999; Chen et al. 2018). The Wanshan Islands together have the same geological origin, and therefore a close biogeographical connection of their atyid faunas is evident.

According to the COI sequence, the range of interspecific genetic divergence (K2P) between *Caridina tetrazona* sp. nov. and the other seven species of *Caridina* was 6.7–32.5%. This result is in accordance with the minimum interspecific genetic distance of 2% recommended by Hebert et al. (2003). The new species is most similar in morphology to *C. serrata* and *C. cantonensis*. According to molecular analyses, the distance between the new species and these two is 0.067 and 0.128, respectively. In the phylogenetic tree, the new species (*C. tetrazona* sp. nov.) can also be separated from *C. cantonensis* and *C. serrata*. Therefore, molecular analyses and morphological analysis are congruent, and together provide sufficient evidence to suggest that the rice shrimp in this study is a new taxon.

As these isolated and vicariant atyid shrimps occur in tightly constrained coastal locations, they may be particularly vulnerable to anthropogenic changes. The island has high potential for ecotourism due to its unspoilt natural landscape and ideal climate. The increase in tourism poses a threat to the survival of this species. Moreover, *Caridina tetrazona* sp. nov. displays a striking coloration pattern, which will certainly receive particular attention among aquarists, so the possible threat by over-harvesting is also present. A program should be developed to guide and control ecological tourism on the island. Monitoring changes in wild populations according to local legislation should also be intensified, and campaigns that promote environmental education and raising tourists’ awareness of the importance of biodiversity should be encouraged.

## Supplementary Material

XML Treatment for Caridina serrata

XML Treatment for Caridina tetrazona
